# Differences in the Transcriptomic Response of *Campylobacter coli* and *Campylobacter lari* to Heat Stress

**DOI:** 10.3389/fmicb.2020.00523

**Published:** 2020-03-27

**Authors:** Carolin Riedel, Konrad U. Förstner, Christoph Püning, Thomas Alter, Cynthia M. Sharma, Greta Gölz

**Affiliations:** ^1^Institute of Food Safety and Food Hygiene, Freie Universität Berlin, Berlin, Germany; ^2^Chair of Molecular Infection Biology II, Institute of Molecular Infection Biology, Julius Maximilian University of Würzburg, Würzburg, Germany; ^3^ZB MED - Information Centre for Life Sciences, Köln, Germany; ^4^Institute of Information Science, Faculty of Information Science and Communication Studies, TH Köln (University of Applied Sciences), Köln, Germany

**Keywords:** *Campylobacter coli*, *Campylobacter lari*, heat stress response, transcriptome sequencing, RNA-seq

## Abstract

*Campylobacter* spp. are one of the most important food-borne pathogens, which are quite susceptible to environmental or technological stressors compared to other zoonotic bacteria. This might be due to the lack of many stress response mechanisms described in other bacteria. Nevertheless, *Campylobacter* is able to survive in the environment and food products. Although some aspects of the heat stress response in *Campylobacter jejuni* are already known, information about the stress response in other *Campylobacter* species are still scarce. In this study, the stress response of *Campylobacter coli* and *Campylobacter lari* to elevated temperatures (46°C) was investigated by survival assays and whole transcriptome analysis. None of the strains survived at 46°C for more than 8 h and approximately 20% of the genes of *C. coli* RM2228 and *C. lari* RM2100 were differentially expressed. The transcriptomic profiles showed enhanced gene expression of several chaperones like *dnaK*, *groES*, *groEL*, and *clpB* in both strains, indicating a general involvement in the heat stress response within the *Campylobacter* species. However, the pronounced differences in the expression pattern between *C. coli* and *C. lari* suggest that stress response mechanisms described for one *Campylobacter* species might be not necessarily transferable to other *Campylobacter* species.

## Introduction

*Campylobacter* is one of the most common causative agents of bacterial food-borne gastroenteritis in humans worldwide. The campylobacteriosis (with clinical symptoms like diarrhea, abdominal cramps, and fever) is mostly self-limiting but sequelae like Guillian–Barré syndrome and reactive arthritis have been described (Poropatich et al., 2010). Handling and consumption of raw or undercooked meat has been identified as a main source for human infections (Alter et al., 2011). *Campylobacter* belongs to the heterogeneous class of *Epsilonproteobacteria* and the species mostly detected in diseased humans are *Campylobacter jejuni* followed by *Campylobacter coli* and to a lesser extent *Campylobacter lari* and *Campylobacter upsaliensis* (Whiley et al., 2013). Whereas *C. jejuni* shows high prevalences in poultry and cattle, *C. coli* is usually associated with pigs and *C. lari* is frequently detected in shellfish and shorebirds (Miller et al., 2008; EFSA and ECDC, 2014). However, in these animals, *Campylobacter* belongs to the commensal microbiota. By comprehensive genome comparisons of several strains, it has been shown that *C. jejuni* is more closely related to *C. coli* compared to *C. lari* (Zhou et al., 2013).

*Campylobacter* spp. possess fastidious growth requirements and are less resistant against environmental and technological stressors compared to other zoonotic pathogens (Park, 2002). This might be explained by the absence of several stress response regulators typically involved in the regulation of stress response to various stressors in other Gram-negative bacteria like *Escherichia coli* or *Salmonella*, e.g., alternative sigma factors like RpoS (σ^38^) as well as other transcription regulators such as CspA, Lrp, SoxRS, and OxyR (Alter and Scherer, 2006). Unlike *E. coli*, which harbors seven sigma factors, only RpoD (σ^70^), RpoN (σ^54^), and RpoF/FliA (σ^28^) were found in *Campylobacter* spp. (Fouts et al., 2005). However, *Campylobacter* spp. is able to survive in the environment and to overcome the barriers along the food chain. This suggests the existence of alternative regulatory mechanisms or a wider role of known regulatory factors. For example, the transcription of both the peroxide as well as the superoxide defense genes are regulated by PerR in *C. jejuni* while in *E. coli* and *Salmonella* OxyR regulates the expression of the peroxide defense regulon and SoxR of the superoxid defense regulon (Kim et al., 2015).

Several authors have described the response of *C. jejuni* to increased temperatures. Konkel et al. (1998) were able to identify 24 proteins preferentially synthesized following heat stress at 46°C. One of these proteins was identified as the DnaJ chaperon, shown to be necessary for survival at elevated temperatures. Further proteome analyses showed 18 differentially expressed proteins induced by increasing temperature from 37 to 42°C (Zhang et al., 2009). The gene expression profiles associated with these responses have also been investigated by microarray analysis (Stintzi, 2003). This study revealed an increased expression of common genes encoding heat stress proteins like chaperones and proteases (e.g., *groELS*, *grpE*, *dnaK*, *dnaJ*, *clpB*, *lon*) and membrane associated proteins (e.g., *galE*, *gmhA2*). In addition, the chaperon activity of the serine protease HtrA is involved in the heat stress response of *C. jejuni* (Baek et al., 2011). The alternative sigma factor σ^32^ (RpoH) mediating the expression of heat stress related genes in *E. coli* is missing in *Campylobacter* (Parkhill et al., 2000; Yura et al., 2000). So far, RacR, HspR, and HrcA have been identified as regulators of the heat stress response in *C. jejuni* (Holmes et al., 2010; Apel et al., 2012), while RpoN (σ^54^), known to be involved in various stress responses, is not involved in the heat stress response of *C. jejuni* (Hwang et al., 2011).

Survival strategies of the related species *C. coli* and *C. lari* at high temperatures are largely unexplored. Thus, our study aimed to compare the effects of heat stress (46°C) on the two *Campylobacter* species *C. coli* and *C. lari* by (i) survival studies and (ii) whole transcriptome (RNA-seq) analyses, to identify genes involved in the heat stress response.

## Materials and Methods

### Bacterial Strains, Media, and Growth Conditions

*Campylobacter* strains were grown on Mueller–Hinton agar containing 5% sheep blood (MHB; OXOID, Wesel, Germany) for 48 h or *Brucella* broth for 24 h (BB; BD, Heidelberg, Germany) at 37°C in microaerobic conditions (6% O_2_, 7% CO_2_, 7% H_2_, 80% N_2_) generated by the Mart Anoxomat system (Drachten, Netherlands). The whole genome sequenced strains *C. coli* RM2228 and *C. lari* RM2100 were selected according to their clinical importance. The *C. coli* strain RM2228, isolated from a chicken carcass, belongs to the phylogenetic clade1, which are mainly responsible for human infections (Sheppard et al., 2010). The *C. lari* strain RM2100 was isolated from a child with watery diarrhea (Miller et al., 2008). Two further field strains of each species were included for survival assays. All strains are listed in Supplementary Table S5.

### Survival Studies

For survival studies, pre-cultures were diluted in BB to an optical density of 0.01 at 600 nm (approximately 7 log_10_ CFU/ml) and incubated at 46°C in microaerobic conditions in a static cultivation mode. The cell numbers were determined over a 24 h period by plating serial dilutions on MHB. These plates were incubated at 37°C for 48 h and cell counts shown as log_10_ CFU/ml. Experiments were performed at three individual time points with technical duplicates.

### Quantitative Real-Time PCR

The expression of the selected genes *clpB*, *grpE*, *dnaK*, *groEL*, *groES*, *cbpA*, and *dnaJ* of *C. coli* and *C. lari* were analyzed over 60 min stress exposure, induced by increasing temperatures from 37°C to 46°C. RNA extractions of three individual cultures were included for both species. Total RNA was isolated from approximately 9 log CFU using a peqGOLD Bacterial RNA Kit (Peqlab, Erlangen, Germany). Removal of genomic DNA was performed in a total volume of 40 μl containing 4 U DNase I, 40 U Ribolock, 1x DNase buffer (all Fermentas, Leon-Rot, Germany), and 28 μl of RNA. After an incubation for 15 min at 37°C, DNase was inactivated by adding 4 μl 50 mM EDTA and heating at 65°C for 10 min. First-strand cDNA was synthesized of 1 μg RNA using the RevertAid Premium First Strand cDNA synthesis kit and random hexamer primers (all Fermentas) according to manufacturer’s instructions.

Primers were designed using the Primer3 web interface^1^ based on the *C. coli* RM2228 and *C. lari* RM2100 genome sequences (with primer length 17–25 nt, amplicon size 60–150 nt, and primer Tm 50–62°C). All runs were performed in a 15 μl PCR mixture containing 1 μl of a 1:10 dilution of cDNA, 0.05–0.9 μM of each primer (Supplementary Table S1) and a twofold SsoFast EvaGreen Supermix (Bio-Rad, Munich, Germany). The amplification was performed by pre-heating for 30 s at 94°C and 40 cycles of 94°C for 5 s followed by annealing for 10 s (annealing temperatures are shown in Supplementary Table S1). Quantitative real-time PCR data were processed using the CFX Manager Software (Bio-Rad) with *thiC* and *rpoA* as housekeeping genes. ΔΔCq values of all samples were determined based on two technical replicates. Specificity of the amplification product was confirmed by melting curve analysis.

### RNA Isolation for Whole Transcriptome Analysis

As quantitative real-time PCR analysis indicated still enhanced mRNA-level for several genes after 30 min heat stress, this time point was chosen for whole transcriptome analysis. RNA was isolated from two independent cultures for each species. Total RNA was extracted from approximately 9 log CFU of the two species *C. coli* and *C. lari* after 30 min cultivation at 37°C and of heat stress at 46°C using a hot phenol/lysozyme method (Blomberg et al., 1990). Therefore, the bacterial culture was mixed 1:5 with stop-mix (95% v/v ethanol, 5% v/v water saturated phenol, −20°C). Cells were pelleted by centrifugation at 1500 × *g*, supernatant discarded, and samples stored at -80°C. Pellets were resuspended in 600 μl 0.5 mg/ml lysozyme in TE (pH 8.0), 60 μl 10% w/v SDS were added and incubated at 64°C for 2 min. Afterward 66 μl 1 M NaOAc (pH 5.2) and 750 μl Roti-Aqua phenol (Carl Roth, Karlsruhe, Germany) were added. During incubation at 64°C for 6 min, samples were mixed by inversion 6–10 times. Tubes were placed on ice and centrifuged for 15 min at 12,000 × *g* (4°C). Aqueous layer was transferred in a 2 ml Phase Lock Gel-Heavy tube (VWR, Dresden, Germany), 750 μl chloroform (Carl Roth) added and mixed by inversion. After centrifugation for 12 min at 12,000 × *g*, the aqueous layer was transferred, 1.4 ml 30:1 EtOh:3 M NaOAc (pH 6.5) added and incubated overnight at −20°C. Samples were centrifuged at 12,000 × *g* (at 4°C for 30 min), supernatant removed, and pellet washed with 900 μl 75% v/v ethanol (-20°C). Samples were centrifuged as mentioned above, ethanol removed, and the pellet air-dried. The RNA was dissolved by adding 100 μl nuclease-free water (Roth) and shaken for 5 min at 65°C (800–1000 r/min). DNA was removed according to the above-described method. RNA integrity and quantity were determined on an Agilent 2100 Bioanalyzer (Agilent Technologies, Waldbronn, Germany) before cDNA synthesis.

### Whole Transcriptome Sequencing

Vertis Biotechnologie AG (Munich, Germany) generated libraries of two independent RNA samples for each condition. The RNA samples were poly(A)-tailed by using poly(A) polymerase. The 5’PPP were removed using tobacco acid pyrophosphatase (TAP) followed by the ligation of the RNA adapter to the 5’-monophosphate of the RNA. First-strand cDNA synthesis was performed with an oligo(dT)-adapter primer and the M-MLV reverse transcriptase. The resulting cDNA was PCR-amplified to reach a concentration of 20–30 ng/μl using a high fidelity DNA polymerase. The cDNA was purified using the Agencourt AMPure XP kit (Beckman Coulter Genomics, Essex, United Kingdom) and was analyzed by capillary electrophoresis. The primers used for PCR amplification were designed for TruSeq sequencing according to the instructions of Illumina (San Diego, CA, United States). The following adapter sequences flank the cDNA inserts: TrueSeq_Sense_primer 5’-AATGATACGGCGACCACCGAGATCTACACTCTTTCCC TACACGACGCTCTTCCGATCT-3’ and TrueSeq_Antisense _NNNNNN_primer (NNNNNN = Barcode) 5’-CAAGCA GAAGACGGCATACGAGATNNNNNNGTGACTGG AGTTC AGACGTGTGCTCTTCCGATC(dT25)-3’. The combined length of the flanking sequences is 146 bases. The libraries were sequenced with an Illumina HiSeq machine with 100 cycles in single end mode.

### Bioinformatical Analysis

#### Read Data Analysis

The resulting sequence reads were demultiplexed and the adapter sequences were removed. After that the reads in Fastq format were quality trimmed using fastq_quality_trimmer (from the FastX suite version 0.0.13^2^) with a quality cut-off score of 20 and converted to fasta format using fastq_to_fasta (also from the FastX suite). The read processing [poly(A) removal, size filtering (min 12 nt length), statistics generation, coverage calculation, and normalization] was performed with the RNA-analysis pipeline READemption version 0.3.4 (Forstner et al., 2014; building upon the following libraries Biopython 1.65, pysam 0.8.1, matplotlib 1.4.3, pandas 0.16.0) which used segemehl version 0.1.7 (Hoffmann et al., 2009) for the read alignment. For *C. coli* RM2228, the sequences with the accession number AAFL01000001.1-38.1, DQ518170, DQ518171, as well as DQ518172.1 and for *C. lari* RM2100 the sequences with the accession number NC_012039 as well as NC_01240.1 were used as references (all mapping statistics are shown in Supplementary Table S2). Feature wise gene-quantification was performed (also with READemption) and used for differential gene expression analysis. According to the study of Stintzi (2003) genes with log_2_ fold changes of expression below -0.58 or above 0.58 and an adjusted (Benjamini–Hochberg corrected) *p*-value < 0.05 calculated by DESeq2 1.6.3 were defined as regulated (Love et al., 2014).

The RNA-Seq data discussed in this publication have been deposited in NCBI’s Gene Expression Omnibus (Edgar et al., 2002) and are accessible through GEO Series accession number GSE67486^3^.

#### Orthologous Mapping

Orthologs were defined, based on the nucleotide sequences, by bidirectional best-BLAST-hit search with max. e-value of 1e-6, word size of 20, and a minimal length of 60% of both query and subject nucleotide sequence. Further some genes were determined as orthologous by similarity of gene name.

#### Functional Grouping

Functional groups of *C. coli*, *C. lari*, and *C. jejuni* CDS were based on functional categories received by eggNOG4.0 database^4^ or by functional category from orthologs. CDS belonging to two or more different categories were listed in each category. Statistical significance of enrichments of functional groups was calculated with Fisher’s exact test using GraphPad Prism version 6.07 for Windows (GraphPad Software, La Jolla, CA, United States^5^).

The bioinformatical analyses represented by Shel and Python scripts as well as their results are deposited at Zenodo^6^.

## Results

### Survival Assays Show High Susceptibility to Heat Stress of *C. lari* and *C. coli*

**FIGURE 1:**
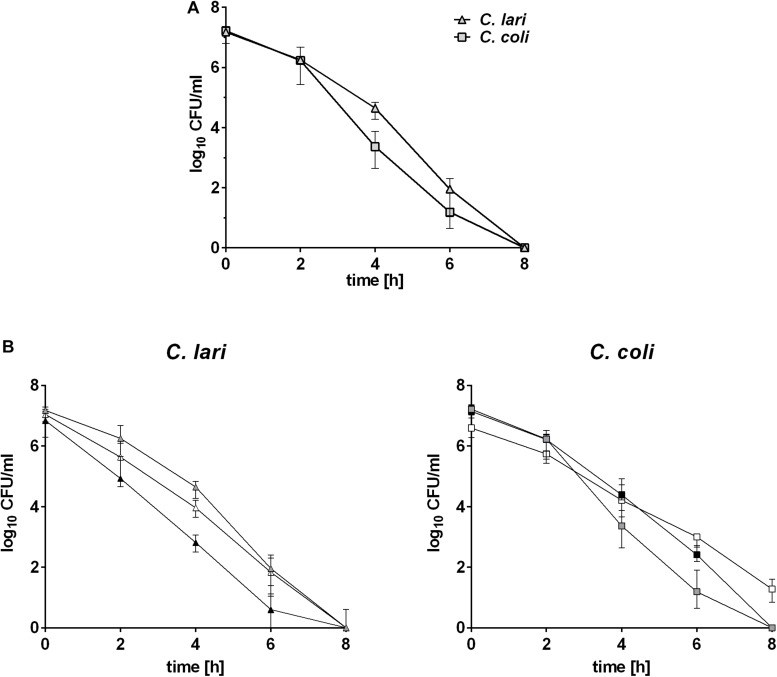
Survival abilities of *C. coli* and *C. lari* at 46°C. Bacterial cultures of approximately 7 log_10_ CFU/ml were incubated at 46°C and cell counts determined at indicated time points. Data were shown for **(A)** reference strains and **(B)** wild type strains of *C. coli* and *C. lari* as median ± interquartile range of three biological replicates. Gray: sequenced strains, white: first field strain, and black: second field strain.

The two strains *C. coli* RM2228 and *C. lari* RM2100 were heat stressed by cultivation at 46°C under microaerobic conditions and survival of these strains was determined over a period of 24 h. Experiments were started with approximately 7 log_10_ CFU/ml (Figure 1A). Cell counts of *C. coli* and *C. lari* were reduced by one log level after 2 h of incubation at 46°C. *C. lari* cell counts decreased to 3.4 log_10_ CFU/ml after 4 h and further 1.1 log_10_ CFU/ml after 6 h of heat stress. The cell counts of *C. coli* were reduced to 4.6 log_10_ CFU/ml after 4 h and further 1.9 log_10_ CFU/ml after 6 h of cultivation at 46°C. Already after 8 h of heat stress, no surviving cells were observed for both strains. To verify that the observed heat stress survival ability is not only strain-specific, the experiments were repeated with two more field isolates of each species (Figure 1B). Even though variations between the isolates were observed, the overall survival was comparable to the results obtained by the laboratory strains shown in Figure 1A.

### Gene Expression Profiling of Known Heat Stress Genes Using qRT-PCR

**FIGURE 2:**
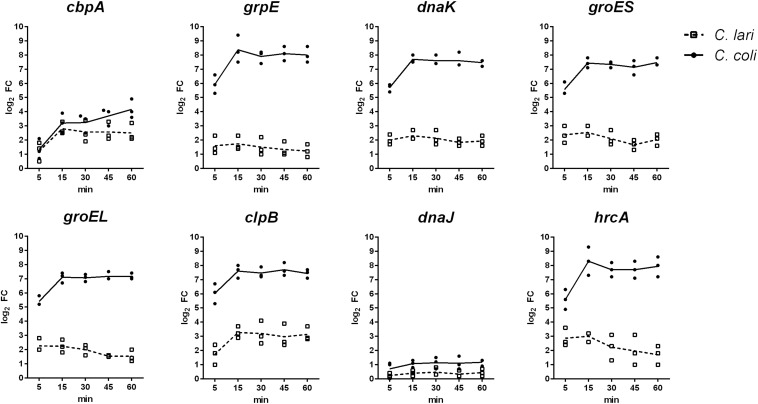
Gene expression of known heat shock genes in *C. coli* RM2228 and *C. lari* RM2100 at 46°C compared to 37°C by qRT-PCR. Gene expression of selected heat shock genes was analyzed by qRT-PCR and normalized to the expression of *rpoA* and *thiC*. Fold changes of expression were calculated by the ΔΔCq method of CFX manager software. Log_2_ fold changes for each biological replicate were shown and the means connected by the line.

The expression level of selected genes, known to be involved in the heat shock response of *C. jejuni*, was investigated over a time period of 60 min of heat stress at 46°C by quantitative RT-PCR in *C. coli* and *C. lari*. In both strains increased log_2_ fold changes of the expression level for the chaperon genes *clpB*, *grpE*, *dnaK*, *groEL*, *groES*, *cbpA* and the negative transcriptional regulator *hrcA* were determined, while expression level of *dnaJ* was only slightly increased in *C. coli* and not regulated in *C. lari* (Figure 2). After 15 min of heat stress, the expression level of these genes was highly induced in both strains. While the high expression levels were stable until the end of the experiments (60 min) in *C. coli*, the levels slightly decreased after 30 min in *C. lari*. Overall, higher fold changes of expression level of these genes were detected in *C. coli* compared to *C. lari*.

### Global Transcriptome Analysis Upon Heat Stress Using RNA-Seq

To get further insight into the heat stress response of both species, whole transcriptome analysis of *C. coli* and *C. lari* was performed by RNA sequencing. Therefore, RNA was extracted for each strain after 30 min cultivation at 37 and 46°C of biological duplicates, libraries prepared as described in Section “Materials and Methods” and transcriptome libraries sequenced by Illumina. Log_2_ fold changes of expression level were determined by DESeq2 (mapping statistics for all libraries are shown in Supplementary Table S2 and log_2_ fold changes of expression level in Supplementary Table S3). The genes already analyzed by qRT-PCR showed similar amplification in the RNA-seq analysis. Expression level of further 18 genes per strain was investigated by qRT-PCR, to correlate the fold changes determined by RNA-seq versus qRT-PCR resulting in a Pearson correlation coefficient *R*^2^ of 0.6644 for *C. coli* and 0.5599 for *C. lari* (Supplementary Figure S1).

**FIGURE 3:**
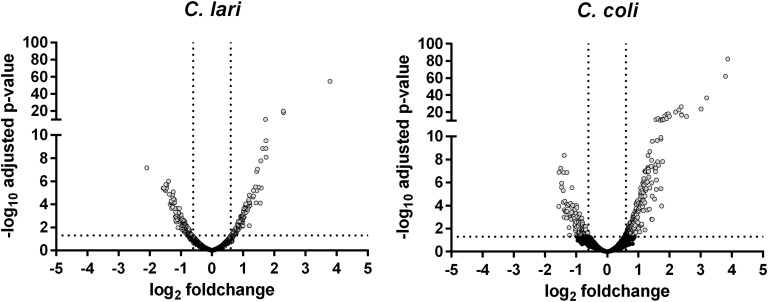
Gene expression profile of the whole transcriptome of *C. coli* RM2228 and *C. lari* RM2100 after 30 min of heat stress. Whole transcriptome was analysis after 30 min incubation at 46°C by RNA sequencing and gene expression shown in volcano plots as log_2_ fold change versus -log_10_ p-values of biological duplicates. Gray: significantly different expressed genes.

After 30 min of heat stress 17.2% of the *C. coli* (338/1967) and 19.4% of the *C. lari* genes (300/1545) were shown by RNA-seq analysis to be differentially expressed (Table 1). Of these differentially expressed genes, 67.1% were up-regulated in *C. coli*, while only 43.6% were up-regulated in *C. lari* (Figure 3).

**TABLE 1:** Gene expression profile by RNA sequencing of *C. coli* RM2228 and *C. lari* RM2100 after 30 min heat stress at 46°C compared to 37°C.

	***C. coli***	***C. lari***
Analyzed genes	1967	1545
Regulated genes	338 (17.2%)	300 (19.4%)
Up-regulated	227 (67.1%)	131 (43.6%)
Down-regulated	111 (32.9%)	169 (56.4%)

*The total numbers of analyzed and differentially expressed genes as calculated by DESeq2 1.6.3 are indicated for each strain.*

### Enrichment of Regulated Genes After Heat Stress in Several Functional Categories

**FIGURE 4:**
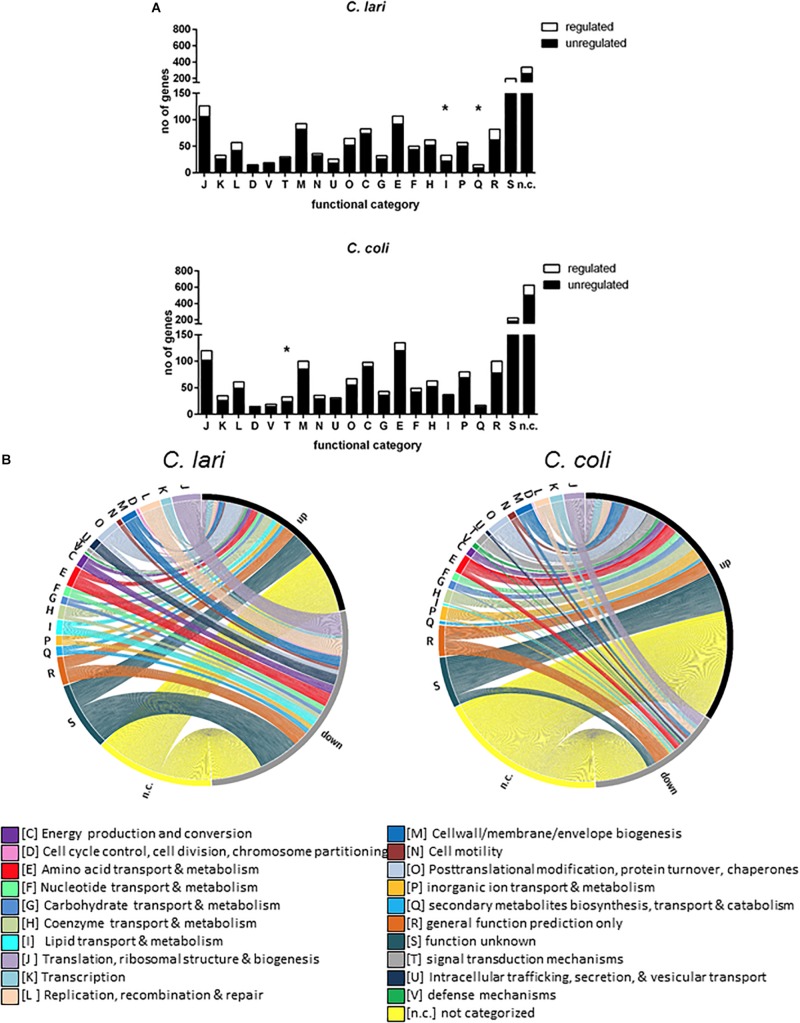
Genes differentially expressed after heat stress according to functional groups. The genes differentially expressed after 30 min of heat stress were shown according to their functional classification (obtained from the evolutionary genealogy of genes: Non-supervised Orthologous Groups http://eggnog.embl.de/version_4.0.beta) for *C. coli* RM2228 and *C. lari* RM2100. **(A)** Total number of regulated and unregulated genes assigned to each functional category were shown for *C. lari* and *C. coli*. Significant enrichment for each category were calculated by Fisher’s exact test (*p < 0.05). **(B)** The figure was created with the open source tool Circos (http://circos.ca). The thickness of each connection displays the log_2_ fold change of gene expression whereas the ending of connection displays if the gene expression is up- or down-regulated.

To analyze if genes with similar function were comparably expressed in both species and if a specific class of genes is significantly enriched upon heat stress, a functional categorization based on orthologous groups according to the eggNOG database was used. Nearly half of the differentially expressed genes (55% for *C. coli* and 49% for *C. lari*) has not been assigned to any category or their functions are only poorly characterized (Supplementary Table S4). The other differentially expressed genes are distributed over the 20 functional categories, with significant enrichment in the category T (signal transduction mechanisms) for *C. coli* (27.3%) and significant enrichment in the category I (lipid transport and metabolism) and category Q (secondary metabolites biosynthesis, transport, and catabolism) for *C. lari* (33.3 and 40%) (Figure 4A).

In both species, the majority of differentially expressed genes in category O (posttranslational modification, protein turn-over, and chaperones) and category H (coenzyme transport and metabolism) were up-regulated, while the majority of differentially expressed genes of category J (translation, ribosomal structure and biogenesis) and U (intracellular trafficking, secretion and vesicular transport) were down-regulated (Figure 4B). Expression level of genes belonging to the category of cell wall/membrane/envelop biosynthesis (M), energy production and conversion (C), and amino acid as well as nucleotide transport and metabolism (E, F) were mostly up-regulated in *C. coli* but down-regulated in *C. lari*. In contrast, nearly no genes belonging to category cell cycle control, cell division, chromosome partitioning (D) or defense mechanisms (V) were affected in their expression level in both species (Supplementary Table S4).

### *C. coli* and *C. lari* Share a Low Number of Differentially Expressed Orthologs Genes With Each Other After Heat Stress

To compare the heat stress response of both species in more detail, orthologs between the genomes of *C. coli* RM2228 and *C. lari* RM2100 were determined. As the knowledge about gene functionality is more comprehensive for *C. jejuni*, we also included the genome of *C. jejuni* NCTC11168 in the ortholog assignment. Altogether 1372 orthologous groups (consisting of two or three genes) could be assigned in the three combined genomes (Supplementary Figure S2) with 759 orthologous genes present in all three genomes. While *C. jejuni* and *C. coli* additionally shared 554 orthologous genes, *C. lari* shared only 18 orthologous genes with *C. coli* and 41 with *C. jejuni*, respectively.

For 70.1% of the 338 regulated genes in *C. coli*, and 57.7% of the 300 regulated genes in *C. lari*, no ortholog could be determined in the other strain. However, the expression of 35 genes, 29 orthologs, and six genes with the same name, but not defined as orthologs at a BLAST e-value of 1e-6 (marked by asterisks in Table 2), was similarly regulated in both species, while 2 genes showed oppositional expression between the two strains (Table 2).

**TABLE 2:** Similar regulated genes after heat stress in *C. coli* RM2228 and *C. lari* RM2100.

**Gene**	**Product**	**Category**	**Locus_tag**	**Log_2_ fc**	***p*adj**	**Locus_tag**	**Log_2_ fc**	***p*adj**
*hrcA*	Heat-inducible transcription repressor	–	Cla_0937	2.30	0.000	CCO0811	3.87	0.000
*pseA*	Pseudaminic acid biosynthesis protein PseA	D	Cla_1328	1.08	0.000	CCO1426	0.75	0.006
*thyX*	FAD-dependent thymidylate synthase	F	Cla_0011	0.87	0.004	CCO0057	0.86	0.003
*gapA*	Glyceraldehyde-3-phosphate dehydrogenase	G	Cla_0417	0.68	0.024	CCO1511	0.68	0.025
*birA*	Biotin–protein ligase	H	Cla_0187	0.64	0.044	CCO1782	1.07	0.000
*ribB*	Bifunctional 3,4-dihydroxy-2-butanone 4-phosphate synthase/GTP cyclohydrolase II protein	H	Cla_0604	1.02	0.001	CCO1384	1.14	0.000
*thrS*	Threonyl-tRNA synthetase	J	Cla_0028	0.85	0.015	CCO0296	1.07	0.000
*hspR*	MerR family transcriptional regulator	K	Cla_0777	1.73	0.000	CCO1318	1.73	0.000
*uvrB*	Excinuclease ABC subunit B	L	Cla_1186	0.69	0.024	CCO0746	1.43	0.000
lolA*	Outer-membrane lipoprotein carrier protein	M	Cla_0524	1.33	0.000	CCO0952	1.02	0.009
flaG*	Flagellar protein FlaG	N	Cla_0765	0.85	0.003	CCO0644	0.98	0.000
*clpB*	Protein disaggregating chaperone ClpB	O	Cla_0711	1.44	0.000	CCO0608	1.92	0.000
*grpE*	Heat shock protein GrpE	O	Cla_0936	1.72	0.000	CCO0812	2.30	0.000
*dnaK*	Molecular chaperone DnaK	O	Cla_0935	1.49	0.000	CCO0813	1.78	0.000
*groES*	Co-chaperonin GroES	O	Cla_1035	1.46	0.000	CCO1296	3.80	0.000
*groEL*	Molecular chaperone GroEL	O	Cla_1034	1.15	0.000	CCO1297	2.00	0.000
*cbpA*	Co-chaperone-curved DNA binding protein A	O	Cla_0778	2.28	0.000	CCO1317	2.38	0.000
*kefB*	Sodium/hydrogen exchanger family protein	P	Cla_0776	1.20	0.000	CCO1319	1.08	0.000
*–*	Multicopper oxidase	Q	Cla_0353	0.87	0.003	CCO1623	0.83	0.002
*–*	Hypothetical protein	S	Cla_1506	1.10	0.000	CCO0311	0.93	0.000
*–*	Hypothetical protein	S	Cla_1010	0.96	0.002	CCO0848	1.14	0.000
*ciaB*	Invasion antigen B	S	Cla_1268	0.63	0.042	CCO1015	0.71	0.030
*plsX*	Glycerol-3-phosphate acyltransferase PlsX	I	Cla_1337	−0.97	0.003	CCO0417	−0.62	0.028
*dusB*	tRNA-dihydrouridine synthase B	J	Cla_0210	−0.71	0.031	CCO1756	−0.83	0.019
*rpmF*	50S ribosomal protein L32	J	Cla_1336	−1.31	0.000	CCO0418	−1.07	0.001
*rpsL*	30S ribosomal protein S12	J	Cla_0451	−0.70	0.034	CCO0588	−0.61	0.022
*queA*	S-adenosylmethionine:tRNA ribosyltransferase-isomerase	J	Cla_0662	−0.73	0.018	CCO1379	−0.71	0.041
*–*	Endoribonuclease L-PSP	J	Cla_0277	−1.24	0.000	CCO1499	−0.87	0.002
cmeD*	Outer membrane component of efflux system	M	Cla_0986	−0.81	0.008	CCO1098	−0.66	0.036
*flgI*	Flagellar basal body P-ring biosynthesis protein FlgI	N	Cla_0400	−0.80	0.012	CCO1569	−0.81	0.001
aat*	Leucyl/phenylalanyl-tRNA–protein transferase	O	Cla_1064	−1.13	0.001	CCO1187	−0.92	0.006
*–*	Hypothetical protein	R	Cla_0749	−0.87	0.004	CCO0785	−1.54	0.000
*–*	Hypothetical protein	S	Cla_1475	−1.25	0.000	CCO1685	−0.69	0.044
ctsE*	Type II protein secretion system E protein CtsE	U	Cla_0392	−0.83	0.015	CCO1577	−0.81	0.021
ctsD*	Type II protein secretion system D protein CtsD	U	Cla_0390	−1.12	0.000	CCO1580	−1.20	0.001
*–*	HIT family hydrolase	FG	Cla_0423	−1.01	0.001	CCO0504	1.24	0.000
*–*	7-Cyano-7-deazaguanine reductase	R	Cla_0006	−1.28	0.000	CCO0022	1.15	0.000

The gene locus, log_2_ fold change of expression, adjusted *p*-value, functional category, as well as the putative gene products of orthologous genes regulated in both species are indicated. *Genes included according to same gene name but not defined as orthologs by BLAST e-value of 1e-6.

Orthologous genes with up-regulated expression levels in both species include the heat stress response related genes *hrcA*, *grpE*, *dnaK*, *groEL*, *groES*, *clpB*, *cbpA*, *hspR*, the invasion antigen *ciaB*, flagellar associated genes *flaG* and *pseA*, membrane associated genes (*lolA*, *kefB*, Cla_1506/CCO0311), the threonyl-tRNA-synthetase *thrS* and the exinuclease *uvrB*.

Orthologous genes down-regulated in both species included translation and ribosomal associated genes (*rpmF*, *rpsL*, *dusB*, *aat*, *queA*, an endoribonuclease), the *Campylobacter* transformation system proteins *ctsE* and *ctsD*, the outer membrane efflux protein *cmeD*, and the flagellar P-ring protein *flgI*.

The expression of the genes encoding a histidine triad protein (Cla_0423/CCO0504) and a GTP cyclohydrolase (Cla_0006/CCO0022) were down-regulated in *C. lari* but up-regulated in *C. coli* (Table 2).

### Genes Affected in Expression After Heat Stress in One Species Only

#### C. coli

Genes assigned to a functional category and with differential expression after heat stress in *C. coli* only are shown in Table 3 (log_2_ fold changes and *p*-values are shown in Supplementary Table S3). The expression of the transcriptional regulators *cmeR*, *furR1* and a regulator belonging to the ArsR-family were also up-regulated in *C. coli*, while expression of CCO1284 (a regulator belonging to the Baf-family) and the transcription termination gene *nusA* were down-regulated. Further, the expression level of several signal transducer or two-component system encoding genes (e.g., *cheV*, the *flgR*-ortholog *rrp-2*, *dccS*) was up-regulated.

**TABLE 3:** Genes differentially regulated after heat stress in *C. coli* RM2228 only.

**Category**	**Up-regulated**	**Down-regulated**
Energy production and conversion	*aspA*, *cydA*, *hydA2*, *ldh*, CCO0259, CCO0482, CCO1660	*fliI*
Amino acid transport and metabolism	*bisZ*, *dapA*, *glnA*, *hisH*, *potA*, *proB*, *serB*, CCO1002, CCO1668	*ilvE*, *proC*, CCO0338, CCO0845, CCO0846, CCO1354, *glnQ*
Nucleotide transport and metabolism	*carA*, *nrdA*, *panC*, *prsA*, *purC*	*surE*
Carbohydrate transport and metabolism	*fucP*, *pgk*, CCO0581 + CCO0582	CCO1026
Coenzyme transport and metabolism	*folC*, *hemH*, *moeA*-3, *panB*, *pdxJ*, *thiL*, CCO1477	*mobB*, CCO1284
Lipid transport and metabolism	*–*	CCO1644
Translation, ribosomal structure, and biogenesis	*cca*, *gatC*, *infC*, *rplS*, *rpsU*, CCO0741	*ksgA*, *prfA*, *rimM*, *rpsL*, CCO0191, CCO0716
Transcription	*cmeR*, *furR1*, *rnr*, CCO0384, CCO1673	*nusA*, CCO0483, CCO0137
Replication, recombination, and repair	*dnaB*, *dnaE*, *recA*, *topA*, *ung*, CCO1639	*dnaQ*, *dprA*, *recG*, *rnhB*, CCO0288
Cell wall/membrane/envelope biogenesis	*lepA*, *lgt*, *murF*, *prc*, *pseF*, *pseG*, *rfaF*, *waaV*, CCO0689, CCO0691	*murB2*, *pbpB*, CCO1291
Cell motility	*flaA*, *flaC*, *cheW*, CCO0943	*flgG_1*, *fliR*
Posttranslational modification, protein turnover, chaperones	*htrA*, *hypC*, CCO0150, CCO1189	*msrA*
Inorganic ion transport and metabolism	*cmeB*, *pstC*, *pstS*, CCO0076, CCO0087, CCO0127 + 28, CCO1232	*cysQ*, *feoB*
Secondary metabolites biosynthesis, transport, and catabolism	CCO0052, CCO1043	–
Signal transduction mechanism	*amt*, *arsC*, *cheV*, *dccS*, *rrp-2*, CCO1327, CCO1600	CCO1314
Intracellular trafficking, secretion, and vesicular transport	CCO1188	–
Defense mechanisms	*cmeA*, *hsdR*, *ybjZ*	CCO0655

*Summary of genes with up- or down-regulated expression level after heat stress and assignment to a functional category for *C. coli* RM2228. The log_2_ fold changes of expression level for each gene are shown in Supplementary Table S3.*

Despite the genes of the translational machinery affected in both species, the expression of the 16S rRNA modification protein (*rimM*) and the translation termination factor *prfA* were also down-regulated in *C. coli*. In contrast, the expression of several ribosomal protein encoding genes as well as the translation initiation factor *infC* were up-regulated.

Further, increased expression levels for the genes *ung* and *recA*, both involved in DNA repair, as well as for the chaperone gene *htrA* have been determined.

In addition, the expression levels of several genes involved in amino acid (e.g., *glnA*, *bisZ, glnQ*, CCO1002), carbohydrate (e.g., *fucP*, CCO0582 + 83), nucleotide, and coenzyme transport and metabolism were up-regulated after the heat shock in *C. coli*.

The expression levels of the majority of genes belonging to the cell wall/membrane/envelope biogenesis (e.g., *pseF*, *pseG*) and the multidrug efflux system encoding genes *cmeA* and *cmeB* were significantly up-regulated whereas the expression of *cmeC* was only slightly increased (Supplementary Table S3).

The expression level of the flagellar filament structure genes *flaA* and *flaC*, and the chemotaxis gene *cheW* were up-regulated. In contrast, genes belonging to secretory function of the flagella like *fliR* and *fliI* as well as the basal body *flgG_1* and the energy delivering protein encoded by *fliI* were down-regulated (Table 3 and Supplementary Table S3).

In contrast, the expression level of other genes involved in energy production and conversion were all up-regulated (Table 3 and Supplementary Table S3).

#### C. lari

Genes assigned to a functional category and with differential expression after heat stress in *C. lari* only are shown in Table 4 (log_2_ fold changes and *p*-values are shown in Supplementary Table S3). The expression of the transcriptional regulator genes Cla_1081 and Cla_0872 was up-regulated while *rpoN* and Cla_0784 (transcriptional regulator of the Crp/Fnr-family) was down-regulated in *C. lari* (Table 4). Of the two-component system or signal transducer encoding genes, a probable *dccS* (Cla_1102) and Cla_1204 were up-regulated, while Cla_0781 was down-regulated.

**TABLE 4:** Genes differentially regulated after heat stress in *C. lari* RM2100 only.

**Category**	**Up-regulated**	**Down-regulated**
Energy production and conversion	*fdxB*, *ndh*	*mez*, *napH*, *oorB*, *petA*, *rdxA*, *torC*
Cell cycle control, cell division, chromosome partitioning	–	*ftsX*
Amino acid transport and metabolism	*pepE*, *selA*, Cla_0593, Cla_0897, Cla_0458	*aroE*, *aspC*, *bioA*, *hisIE*, *livH*, *modC*, *pglE*, *sdaA*, *sdaC*, *selD*
Nucleotide transport and metabolism	*prsA*	*purE*, *pyrE*, Cla_0004
Carbohydrate transport and metabolism	*rpe*, Cla_1277	*tal*, Cla_1361
Coenzyme transport and metabolism	*mobA*, *ribD*, *thiD*, *thiM*, Cla_0686	*dxs*, *folP*, *hemC*
Lipid transport and metabolism	*aas*, *cdsA*, *dxr*, *fabZ*	*fabD*, *fabH3*, *ispE*, *pgpA*, *pgsA*, Cla_1314
Translation, ribosomal structure, and biogenesis	*prfB*, *truB*, Cla_0258	*gltX*, *leuS*, *rbfA*, *rplE*, *rplF*, *rplJ*, *rplX*, *tlyA*, *truA*, Cla_0017, Cla_1325
Transcription	Cla_0872, Cla_1081, Cla_1538	*npdA*, *rpoN*, Cla_0784
Replication, recombination, and repair	*xth*, Cla_0824	*dnaX*, *ligA*, *ligA-2*, *mutS*, *nth*, *rep*, *ruvA*, *uvrC*, Cla_0401, Cla_0671, Cla_0836, Cla_0945
Cell wall/membrane/envelope biogenesis	*pseB*, *ptmB*, Cla_0217	*kdsB*, *murC*, *mreC*, *pbpA*, *pglF*, Cla_0642
Cell motility	*–*	*flgG*, *fliN*
Posttranslational modification, protein turnover, chaperones	*dnaJ*, Cla_0589 + 90, Cla_1212	*hypA*, Cla_1116
Inorganic ion transport and metabolism	*modB*, *cft*	*cmeF*, Cla_0159, Cla_0643, Cla_0109
Secondary metabolites biosynthesis, transport, and catabolism	Cla_0039, Cla_0565	*iamA*, *iamb*, Cla_0207
Signal transduction mechanism	Cla_1102, Cla_1204	Cla_0781
Intracellular trafficking, secretion, and vesicular transport	–	*ctsG*, *exbD1*, *exbB1*, *secF*, *secY*, Cla_0954
Defense mechanisms	*kpsM*	Cla_1528

*Summary of genes with up- or down-regulated expression level after heat stress and assignment to a functional category for *C. lari* RM2100. The log_2_ fold changes of expression level for each gene are shown in Supplementary Table S3.*

Even though decreased expression levels for the majority of genes belonging to the translational machinery were determined, the expression level of *prfB*, involved in termination of translation, and Cla_0258, a ribosomal modification protein, were up-regulated (Table 4 and Supplementary Table S3).

The expression of the chaperon genes *dnaJ*, the DnaJ domain protein encoding gene Cla_0590, as well as *xth* (involved in DNA repair) was up-regulated. However, several other genes also involved in DNA repair and replication were down-regulated (Table 4 and Supplementary Table S3).

A significant enrichment in the category of lipid transport and metabolism has been determined and the majority of the differentially expressed genes were down-regulated.

The expression of genes involved in LOS biosynthesis (*kdsB*), rod shape formation (*mreC*), peptidoglycan biosynthesis (*pbpA*, *murC*), or general cell wall/membrane/envelop biogenesis (*pglF* and Cla_0642) were down-regulated in *C. lari*.

The flagellin modification genes *ptmB* and *pseB* were up-regulated, while the flagellar motor switch protein encoding gene *fliN*, and the basal body rod protein encoding gene *flgG* were down-regulated.

## Discussion

It has previously been shown that *Campylobacter* spp. are rather sensitive to elevated temperatures. Baserisalehi et al. (2006) determined similar D-values for *C. coli* and *C. lari* in food matrices at elevated temperatures. Our data showed that *C. coli* and *C. lari* are similarly susceptible to heat stress at 46°C in *Brucella* broth, too. In contrast to *C. jejuni*, studies about the heat shock response of these two *Campylobacter* species on mRNA or protein level were missing so far. Therefore, we analyzed changes in the whole transcriptome of the two strains after heat stress. The overall percentage of differentially expressed genes was comparable between *C. coli* (17.2%) and *C. lari* (19.4%). However, the majority of differentially expressed genes were up-regulated in *C. coli* (67.1%) but down-regulated in *C. lari* (56.4%), indicating a different heat stress response of the two species.

Of the overall transcriptional changes detected, only 35 genes were similarly expressed indicating a general role in heat stress response of *Campylobacter* spp. This group includes the transcriptional regulators *hspR* and *hrcA* as well as the chaperons *grpE*, *dnaK*, *groEL*, *groES*, *clpB*, and *cbpA.* These genes are included in the 30 genes whose expression has been most highly up-regulated in both species (Supplementary Table S3), and are also involved in the heat stress response of *C. jejuni* (Stintzi, 2003; Holmes et al., 2010). Stintzi (2003) described a high and rapid up-regulation of these genes in *C. jejuni* after temperature increase from 37 to 42°C. This up-regulation of gene expression was only transient, as expression levels nearly reached basal levels after 50 min of temperature up-shift indicating that adaptation to the new conditions was achieved (Stintzi, 2003). As we did not observe any growth at 46°C for the strains investigated in our study, we did not expect adaptation to this conditions, and therefore also no transient changes in the gene expression pattern. This was also supported by the observation that known heat shock genes were still highly expressed after 60 min of heat stress in our study (Figure 2).

Regarding chaperone DnaJ, Konkel et al. (1998) showed higher protein levels after heat stress for *C. jejuni*. In our study, the expression pattern for *dnaJ* was contradictory. Slightly increased expression of *dnaJ* has been determined by RT-PCR for *C. coli* and by RNA-seq for *C. lari* only. However, Holmes et al. (2010) suggested that CbpA, showing 44% similarity to DnaJ, is the main DnaJ protein in *C. jejuni*. In concordance with that, we observed significantly up-regulated expression of *cbpA* in *C. coli* and *C. lari* in both assays. The only gene belonging to the category of posttranslational modification, protein turnover, chaperons down-regulated in both species was *aat*. The protein Aat might be involved in N-end rule pathway of protein degradation, supposing changes in half-life time of several proteins (Varshavsky, 2011).

The periplasmic chaperone HtrA, which degrades and prevents aggregation of misfolded periplasmic proteins, has been described to be essential for *C. jejuni* growth at 44°C (Baek et al., 2011). Increased expression of *htrA* was also observed in *C. coli* but not in *C. lari*, suggesting that other chaperons or proteases replace the activity of HtrA in *C. lari*. Interestingly, also *racR* was not differentially expressed in *C. coli* and *C. lari*, indicating further regulatory differences compared to the heat shock response of *C. jejuni* (Apel et al., 2012).

Regarding the ribosomal genes, Stintzi (2003) described a transient down-regulated expression in *C. jejuni* after temperature increase from 37 to 42°C. The expression of these genes decreased until 20 min and nearly reached baseline level at 50 min after temperature increase, suggesting that *C. jejuni* transiently reshuffled energy for stress damage repair and adaptation to the new growth condition (Stintzi, 2003). Similarly, the majority of differentially expressed genes assigned to the category of translation and ribosomal structure was also down-regulated in *C. coli* and in *C. lari* at 30 min after heat stress of 46°C. Also comparable to the observations described for *C. jejuni* (Stintzi, 2003), the majority of all differentially expressed genes was up-regulated in *C. coli*, implying high energy costs of heat stress response. According to this hypothesis, the expression of the majority of genes involved in energy metabolism was also up-regulated in *C. coli*. In contrast, the majority of all differentially expressed genes (including genes assigned to the category of energy metabolism) were down-regulated in *C. lari*, suggesting a divergent energy saving behavior compared to *C. coli* under the heat stress condition investigated in our study. The differences in energy metabolism is further supported by the fact that multiple genes involved in energy metabolism and respiration in *C. jejuni* are missing in *C. lari* (Miller et al., 2008, 2014). Nevertheless, the question which transcriptional changes would be further transferred to protein level remains unanswered.

Additional regulators seem to be involved in the heat stress response in both strains tested. While *C. coli* also up-regulated the expression of the transcriptional regulator *fur1*, the ortholog *perR* was not regulated in *C. lari*. PerR has been shown to regulate the expression of at least 104 genes in *C. jejuni*, most of them involved in oxidative stress response (Palyada et al., 2009). Further, *perR* expression is up-regulated after acid stress (Reid et al., 2008). Our data could indicate a further role of PerR in the heat stress response of *C. coli* but not of *C. lari.*

The two-component system DccRS is required for initial adaptation of *C. jejuni* to the gastrointestinal milieu of chickens and controls a regulon of six genes annotated as putative periplasmic or membrane proteins, and *dccR* itself (Wosten et al., 2010). The expression of the putative sensor *dccS* (CCO1300) was up-regulated after heat stress in *C. coli*. In concordance, six genes defined as orthologs or annotated as probably *C. jejuni* genes of the DccRS regulon (CCO0021, CCO0198, CCO0689, CCO0290, CCO1066, CCO1462) were up-regulated in *C. coli*. For *C. jejuni*, it has been demonstrated that neither temperature of 42°C nor copper but growth phase is responsible for activation of DccRS (Wosten et al., 2010). However, our data suggest that the DccRS system is also involved in the heat stress response of *C. coli* at 46°C.

Likewise to *C. coli*, the expression of further possible transcriptional regulators was regulated in *C. lari*. The highest up-regulated expression in *C. lari* was observed for Cla_0805, encoding a peptidase S24 family protein, predicted as transcriptional repressors of the SOS-response in many bacteria (Butala et al., 2009). Interestingly, many of the SOS-response genes described in other bacteria, including the peptidase S24 LexA, are lacking in *C. jejuni* and no ortholog has been detected in *C. coli* (Zgur-Bertok, 2013). Whether *C. lari* has a complete SOS-response has to be elucidated in further studies.

Further changes observed after heat stress include metabolism pathways. Glutamine is the major nitrogen donor for *C. jejuni* and *C. jejuni* 81-176, mutated in the glutamine ABC-Transporter (Cj0469), was more susceptible to heat stress compared to the wild type (Lin et al., 2009). Further, *C. jejuni* use glutamine as a carbon source (van der Hooft et al., 2018). We determined enhanced gene expression of genes involved in glutamine metabolism for *C. coli*, like the degenerated ammonium transporter (CCO0599), the glutamine synthetase *glnA*, the glutamine transporter ATPase *glnQ*, as well as the adjacent located amino acid transporter permease (CCO1002).

Recently, an energy metabolism pathway involved in the formation of pyruvate and L-lactate from L-fucose has been described for *C. jejuni* (Stahl et al., 2012). These genes are located on a genomic island spanning from Cj0480-89 and determined as ortholog for *C. coli* RM2228 by our comparison (CCO0578-87) (Muraoka and Zhang, 2011; Stahl et al., 2011). As the expression of these genes was up-regulated, we suggest that the activation of nitrogen assimilation and the L-fucose metabolic pathway is part of heat stress response in *C. coli* RM2228.

Many genes assigned to the functional category of cell wall/membrane/envelope biogenesis and hypothetical membrane proteins were differentially expressed in *C. coli* and *C. lari*. However, the expression of the majority of these genes was up-regulated by *C. coli* and down-regulated by *C. lari* suggesting different modifications of cell wall/membrane proteins after heat stress in both species.

## Conclusion

Despite similar phenotypic survival abilities at 46°C, distinct variations in the transcriptomic response to heat stress could be determined for *C. coli* and *C. lari*. Similar expression changes for chaperone genes after heat stress, which have also been described for *C. jejuni*, indicate a general response mechanism of *Campylobacter* species. However, the differences in expression of transcriptional regulators and metabolism related genes observed between the tested *C. coli* and *C. lari* strains could be explained in part by the phylogenetic distance and therefore the large differences in the genomic content of these two species. Higher similarities have been determined for the heat stress response of *C. coli* with already published data for *C. jejuni*. This is in agreement with the higher amount of orthologous genes determined for *C. jejuni* and *C. coli* compared to *C. lari*.

Overall, the described transcriptomic changes induced after heat stress in *C. coli* and *C. lari* indicate huge differences in the heat stress response between the *Campylobacter* species.

## Data Availability Statement

The datasets generated for this study can be found in the GEO Series accession number GSE67486 (www.ncbi.nlm.nih.gov/geo/query/acc.cgi?acc=GSE67486).

## Author Contributions

GG and TA planned the study. CR and CP performed the experiments in this study. KF and CS performed the RNA sequencing and bioinformatic analyses. CR, TA, and GG analyzed results, formatted the data, and drafted the manuscript. TA provided funding. All authors read and approved the final manuscript.

## Conflict of Interest

The authors declare that the research was conducted in the absence of any commercial or financial relationships that could be construed as a potential conflict of interest.

## Footnotes

1 http://frodo.wi.mit.edu/

2 http://hannonlab.cshl.edu/fastx_toolkit/

3 http://www.ncbi.nlm.nih.gov/geo/query/acc.cgi?acc = GSE67486

4 http://eggnog.embl.de/version_4.0.beta/downloads.v4.html

5 http://www.graphpad.com

6 https://doi.org/10.5281/zenodo.3669112

## Supplementary Material

FIGURE S1 Correlation of log_2_ fold changes of gene expression by qRT-PCR and RNA-seq. The correlation of the log*_2_* fold changes of selected gene expression after 30 min of heat stress determined by RNA-seq (*x*-axis) and qRT-PCR (*y*-axis) is depicted with corresponding *R*^2^ values for *C. coli* RM2228 and *C. lari* RM2100.

FIGURE S2 Orthologous mapping of the complete genomes from *C. coli* RM2228, *C. lari* RM2100, and *C. jejuni* NCzTC11168. Orthologous were defined by bidirectional best BLAST-hit search on nucleotide level with max. e-value of 1e-6, word size of 20, and a minimal length of 60%. The Venn diagram shows orthologous gene shared by the *C. coli* strain RM2228, *C. lari* strain RM2100, and *C. jejuni* strain NCTC11168.

TABLE S1 Primers used in this study. The target gene, primer sequence and concentration, the amplicon length, and annealing temperature of each primer used for qRT-PCR for both strains are indicated in this table.

TABLE S2 Mapping statistics for all libraries in the RNA-seq. This table indicates the total number of sequenced cDNA reads considered in the analysis and used for alignment, the total number of aligned and of uniquely aligned reads, the total number of alignments, and the percentage of aligned and uniquely aligned reads for the replicates at 37 and 46°C for both strains.

TABLE S3 Excel file containing the Orthologs of differentially regulated genes after heat stress in *C. coli* RM2228 and *C. lari* RM2100. All genes for which a significant regulation of expression was calculated by DESeq2 1.6.3 after 30 min heat stress by RNA-seq analysis were indicated in this table. For each gene, the locus_tag, gene name, gene product, log_2_ fold change of gene expression with adjusted *p*-value, assignment to functional category by eggnog, and their corresponding ortholog are listed. Gene names in parentheses were transferred from orthologs. -: no gene name, function, or category assigned.

TABLE S4 Percentage of differentially expressed genes after heat stress according to eggNOG functional categories. The table indicates the percentage of regulated genes for each strain and category. Genes assigned in two categories were listed in each category. Significant enrichment was calculated by Fisher’s exact test. ^∗^*p* < 0.05.

TABLE S5 Strains used in this study. The ID, origin, description, and source of each strain used in this study are indicated in this table.
