# Supplementary material for: Differences in the Transcriptomic Response of Campylobacter coli and Campylobacter lari to Heat Stress
Source: Front Microbiol. 2020 Mar 27;11:523. doi: 10.3389/fmicb.2020.00523 (PMC7118207; doi:10.3389/fmicb.2020.00523)
Supplement: FIGURE S1 — Correlation of log2 fold changes of gene expression by qRT-PCR and RNA-seq. The correlation of the log2 fold changes of selected gene expression after 30 min of heat stress determined by RNA-seq (x-axis) and qRT-PCR (y-axis) is depicted with corresponding R2 values for C. coli RM2228 and C. lari RM2100. [file Image_1.pdf]

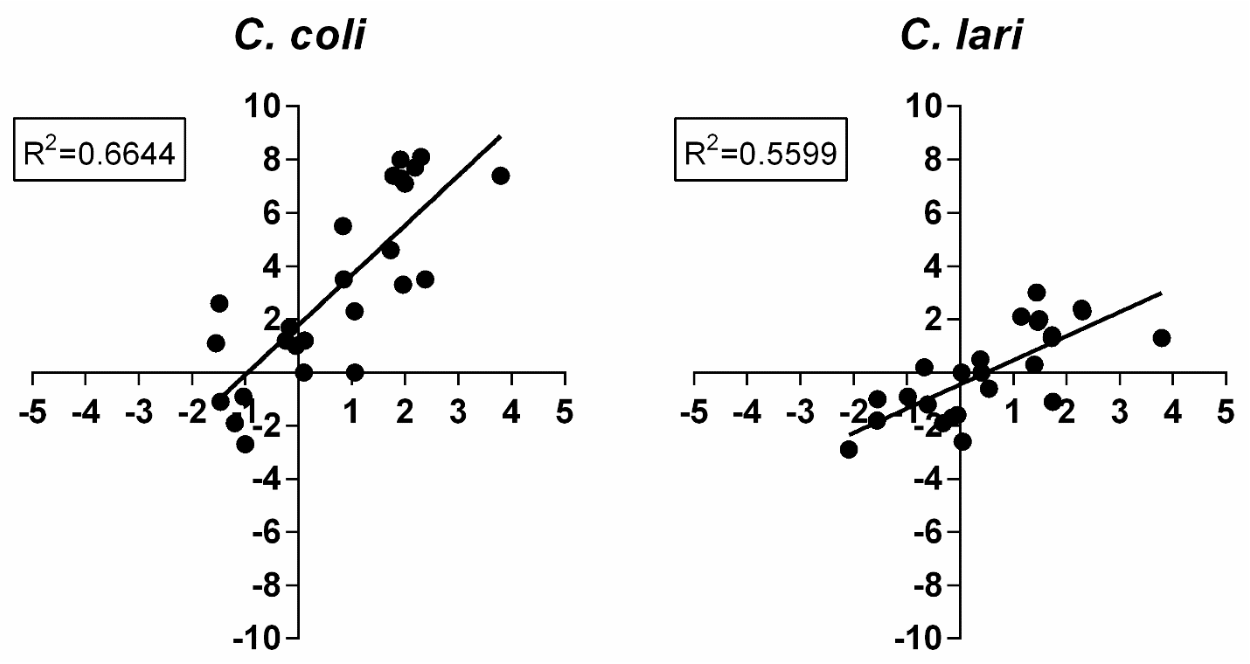

Figure S1: Correlation of log_2 fold changes of gene expression by qRT-PCR and RNA-seq. The correlation of the log_2 fold changes of selected gene expression after 30 min of heat stress determined by RNA-seq (x-axis) and qRT-PCR (y-axis) is depicted with corresponding R^2 values for *C. coli* RM2228 and *C. lari* RM2100.
