# Supplementary figures and images for: Differences in the Transcriptomic Response of Campylobacter coli and Campylobacter lari to Heat Stress
Source: Front Microbiol. 2020 Mar 27;11:523. doi: 10.3389/fmicb.2020.00523 (PMC7118207; doi:10.3389/fmicb.2020.00523)

*C. coli* (2035)

*C. jejuni* (1663)

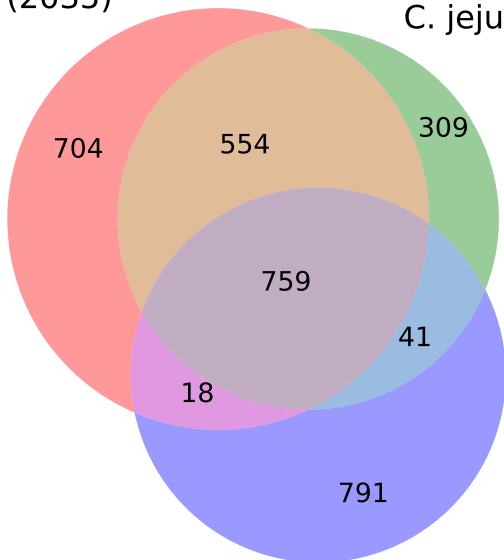

*C. lari* (1609)

Supplement: FIGURE S2 — Orthologous mapping of the complete genomes from C. coli RM2228, C. lari RM2100, and C. jejuni NCzTC11168. Orthologous were defined by bidirectional best BLAST-hit search on nucleotide level with max. e-value of 1e-6, word size of 20, and a minimal length of 60%. The Venn diagram shows orthologous gene shared by the C. coli strain RM2228, C. lari strain RM2100, and C. jejuni strain NCTC11168. [file Image_2.pdf]
