# Supplementary material for: Differences in the Transcriptomic Response of Campylobacter coli and Campylobacter lari to Heat Stress
Source: Front Microbiol. 2020 Mar 27;11:523. doi: 10.3389/fmicb.2020.00523 (PMC7118207; doi:10.3389/fmicb.2020.00523)
Supplement: TABLE S2 — Mapping statistics for all libraries in the RNA-seq. This table indicates the total number of sequenced cDNA reads considered in the analysis and used for alignment, the total number of aligned and of uniquely aligned reads, the total number of alignments, and the percentage of aligned and uniquely aligned reads for the replicates at 37 and 46°C for both strains. [file Table_2.DOCX]

**Table S2: Mapping statistics for all libraries in the RNA-seq.**

This table indicates the total number of sequenced cDNA reads considered in the analysis and used for alignment, the total number of aligned and of uniquely aligned reads, the total number of alignments and the percentage of aligned and uniquely aligned reads for the replicates at 37°C and 46°C for both strains.

|  | *C. coli* RM2228 | | | | *C. lari* RM2100 | | | |
| --- | --- | --- | --- | --- | --- | --- | --- | --- |
| Libraries | 37°C_A | 37°C_B | 46°C_A | 46°C_B | 37°C_A | 37°C_B | 46°C_A | 46°C_B |
| No. of input reads | 6272544 | 5062453 | 6020526 | 3913210 | 9029227 | 8037586 | 9393521 | 6112238 |
| No. of reads - long enough and used for alignment | 6210299 | 4896098 | 5974104 | 3756369 | 8920861 | 7754411 | 9221859 | 5918361 |
| Total no. of aligned reads | 5735964 | 4446165 | 5401631 | 3525425 | 8738925 | 7443242 | 9020797 | 5753029 |
| Total no. of uniquely aligned reads | 3244013 | 2480299 | 3322105 | 1946139 | 5472475 | 5079677 | 5614072 | 3664875 |
| Total no. of alignments | 10727335 | 8409791 | 9587327 | 6698775 | 15007777 | 11946951 | 15546974 | 9753774 |
| % of aligned reads (compared to no. of long enough reads) | 91.45 | 90.81 | 89.72 | 93.85 | 97.96 | 95.99 | 97.82 | 97.21 |
| % of uniquely aligned reads (in relation to all aligned reads) | 56.56 | 55.79 | 61.5 | 55.2 | 62.62 | 68.25 | 62.23 | 63.7 |
