# Supplementary material for: Differences in the Transcriptomic Response of Campylobacter coli and Campylobacter lari to Heat Stress
Source: Front Microbiol. 2020 Mar 27;11:523. doi: 10.3389/fmicb.2020.00523 (PMC7118207; doi:10.3389/fmicb.2020.00523)
Supplement: TABLE S4 — Percentage of differentially expressed genes after heat stress according to eggNOG functional categories. The table indicates the percentage of regulated genes for each strain and category. Genes assigned in two categories were listed in each category. Significant enrichment was calculated by Fisher’s exact test. ∗p < 0.05. [file Table_4.DOCX]

**Table S4: Percentage of differentially expressed genes after heat stress according to eggNOG functional categories.**

The table indicates the percentage of regulated genes for each strain and category. Genes assigned in two categories were listed in each category. Significant enrichment was calculated by Fisher’s exact Test. * = p < 0.05

| functional category | *C. coli* | | | *C. lari* | |
| --- | --- | --- | --- | --- | --- |
|  | in % | (reg/total) | in % | | (reg/total) |
| [C] Energy production and conversion | 8.2 | (8/98) | 10.8 | | (9/83) |
| [D] Cell cycle control, cell division, chromosome partitioning | 6.7 | (1/15) | 13.3 | | (2/15) |
| [E] Amino acid transport and metabolism | 11.1 | (15/135) | 14.0 | | (15/107) |
| [F] Nucleotide transport and metabolism | 14.3 | (7/49) | 12.0 | | (6/50) |
| [G] Carbohydrate transport and metabolism | 16.3 | (7/43) | 18.8 | | (6/32) |
| [H] Coenzyme transport and metabolism | 17.5 | (11/63) | 16.1 | | (10/62) |
| [I] Lipid transport and metabolism | 5.4 | (2/37) | **33.3*** | | (11/33) |
| [J] Translation, ribosomal structure and biogenesis | 15.0 | (18/120) | 15.9 | | (20/126) |
| [K] Transcription | 25.7 | (9/35) | 21.2 | | (7/33) |
| [L] Replication, recombination and repair | 19.7 | (12/61) | 26.3 | | (15/57) |
| [M] Cell wall/membrane/envelope biogenesis | 15.0 | (15/100) | 11.8 | | (11/93) |
| [N] Cell motility | 19.4 | (7/36) | 11.1 | | (4/36) |
| [O] Posttranslational modification, protein turnover, chaperones | 17.9 | (12/67) | 20.0 | | (13/65) |
| [P] Inorganic ion transport and metabolism | 13.8 | (11/80) | 12.3 | | (7/57) |
| [Q] Secondary metabolites biosynthesis, transport and catabolism | 17.6 | (3/17) | **40*** | | (6/15) |
| [T] Signal transduction mechanisms | **27.3*** | (9/33) | 10.0 | | (3/30) |
| [U] Intracellular trafficking, secretion, and vesicular transport | 9.7 | (3/31) | 30.8 | | (8/26) |
| [V] Defense mechanisms | 21.1 | (4/19) | 10.5 | | (2/19) |
| [R] General function prediction only |  | (22/100) |  | | (20/82) |
| [S] Function unknown |  | (40/225) |  | | (49/199) |
| not categorized |  | (124/627) |  | | (77/336) |
