# Supplementary material for: Differences in the Transcriptomic Response of Campylobacter coli and Campylobacter lari to Heat Stress
Source: Front Microbiol. 2020 Mar 27;11:523. doi: 10.3389/fmicb.2020.00523 (PMC7118207; doi:10.3389/fmicb.2020.00523)
Supplement: TABLE S5 — Strains used in this study. The ID, origin, description, and source of each strain used in this study are indicated in this table. [file Table_5.DOCX]

**Table S5: Strains used in this study.**

The ID, origin, description and source of each strain used in this study are indicated in this table.

| Species | Strain ID | Origin | Description | Source |
| --- | --- | --- | --- | --- |
| *C. coli* | RM2228 | poultry | genome sequenced strain | American Type Culture Collection (ATCC) |
|  | ILH 250 | poultry | field strain | our strain collection |
|  | ILH 651 | pig | field strain | our strain collection |
| *C. lari* | RM2100 | human | genome sequenced strain | American Type Culture Collection (ATCC) |
|  | ILH 494 | mussel | field strain | our strain collection |
|  | ILH 496 | mussel | field strain | our strain collection |
